# Cost-Effectiveness of Universal Asymptomatic Preoperative SARS-CoV-2 Polymerase Chain Reaction Screening: A Cost-Utility Analysis

**DOI:** 10.1093/cid/ciad463

**Published:** 2023-08-09

**Authors:** Shunsuke Uno, Rei Goto, Kimiko Honda, Sho Uchida, Yoshifumi Uwamino, Ho Namkoong, Ayumi Yoshifuji, Kei Mikita, Yaoko Takano, Morio Matsumoto, Yuko Kitagawa, Naoki Hasegawa

**Affiliations:** Department of Infectious Diseases, Keio University School of Medicine, Tokyo, Japan; Health Technology Assessment Unit, Department of Preventive Medicine and Public Health, Keio University School of Medicine, Tokyo, Japan; Health Technology Assessment Unit, Department of Preventive Medicine and Public Health, Keio University School of Medicine, Tokyo, Japan; Graduate School of Business Administration, Keio University, Kanagawa, Japan; Graduate School of Health Management, Keio University, Kanagawa, Japan; Health Technology Assessment Unit, Department of Preventive Medicine and Public Health, Keio University School of Medicine, Tokyo, Japan; Graduate School of Health Management, Keio University, Kanagawa, Japan; Center of Health Economics and Health Technology Assessment, Keio University Global Research Institute, Tokyo, Japan; Department of Infectious Diseases, Keio University School of Medicine, Tokyo, Japan; Department of Laboratory Medicine, Keio University School of Medicine, Tokyo, Japan; Department of Infectious Diseases, Keio University School of Medicine, Tokyo, Japan; Department of Infectious Diseases, Keio University School of Medicine, Tokyo, Japan; Department of Infectious Diseases, Keio University School of Medicine, Tokyo, Japan; Division of Infectious Diseases and Infection Control, Keio University Hospital, Tokyo, Japan; Department of Orthopedics, Keio University School of Medicine, Tokyo, Japan; Department of Surgery, Keio University School of Medicine, Tokyo, Japan; Department of Infectious Diseases, Keio University School of Medicine, Tokyo, Japan

**Keywords:** universal asymptomatic screening, preoperative, cost-effectiveness, COVID-19, polymerase chain reaction

## Abstract

**Background:**

An early report has shown the clinical benefit of the asymptomatic preoperative severe acute respiratory syndrome coronavirus 2 (SARS-CoV-2) screening test, and some clinical guidelines recommended this test. However, the cost-effectiveness of asymptomatic screening was not evaluated. We aimed to investigate the cost-effectiveness of universal preoperative screening of asymptomatic patients for SARS-CoV-2 using polymerase chain reaction (PCR) testing.

**Methods:**

We evaluated the cost-effectiveness of asymptomatic screening using a decision tree model from a payer perspective, assuming that the test-positive rate was 0.07% and the screening cost was 8500 Japanese yen (JPY) (approximately 7601 US dollars [USD]). The input parameter was derived from the available evidence reported in the literature. A willingness-to-pay threshold was set at 5 000 000 JPY/quality-adjusted life-year (QALY).

**Results:**

The incremental cost of 1 death averted was 74 469 236 JPY (approximately 566 048 USD) and 291 123 368 JPY/QALY (approximately 2 212 856 USD/QALY), which was above the 5 000 000 JPY/QALY willingness-to-pay threshold. The incremental cost-effectiveness ratio fell below 5 000 000 JPY/QALY only when the test-positive rate exceeded 0.739%. However, when the probability of developing a postoperative pulmonary complication among SARS-CoV-2–positive patients was below 0.22, asymptomatic screening was never cost-effective, regardless of how high the test-positive rate became.

**Conclusions:**

Asymptomatic preoperative universal SARS-CoV-2 PCR screening is not cost-effective in the base case analysis. The cost-effectiveness mainly depends on the test-positive rate, the frequency of postoperative pulmonary complications, and the screening costs; however, no matter how high the test-positive rate, the cost-effectiveness is poor if the probability of developing postoperative pulmonary complications among patients positive for SARS-CoV-2 is sufficiently reduced.

## Teaser

In the base case analysis, asymptomatic preoperative universal SARS-CoV-2 PCR screening is not cost-effective. The cost-effectiveness depends mainly on the test-positive rate, the frequency of postoperative pulmonary complications, and screening costs.

## Graphical Abstract

**Graphical Abstract:**
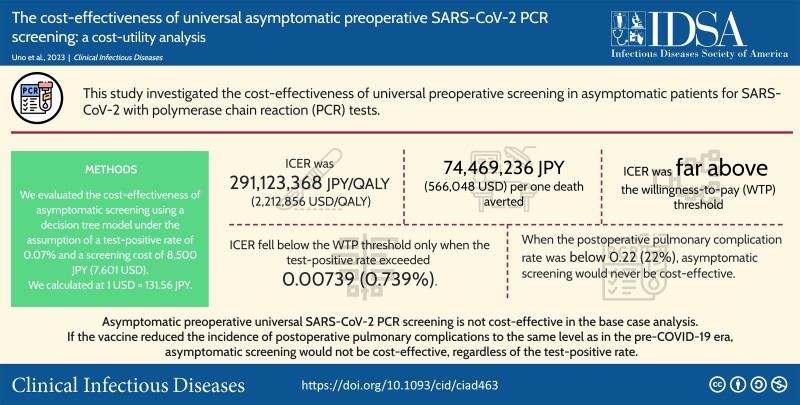
This graphical abstract is also available at Tidbit: https://tidbitapp.io/tidbits/the-cost-effectiveness-of-universal-asymptomatic-preoperative-sars-cov-2-pcr-screening-a-cost-utility-analysis-c17c1267-0831-4c25-ac98-821a6ccdeb3c

The emergence of severe acute respiratory syndrome coronavirus 2 (SARS-CoV-2) led to the global coronavirus disease 2019 (COVID-19) pandemic, which profoundly affected healthcare systems worldwide. The pandemic has significantly impacted surgical procedures, causing delays or cancellations [1] and postoperative pulmonary complications (PPCs) [2, 3]. The World Health Organization has declared that the public health emergency of international concern due to COVID-19 has ended [4]. Therefore, the key issue is how to mitigate the measures based on evidence that, thus far, have been taken on an emergency basis.

With some early reports of a high risk of postoperative morbidity and death in patients exposed to COVID-19 at the time of surgery [5, 6], some guidelines recommend implementing preoperative nucleic acid amplification testing strategies [7, 8] to minimize the risk of postoperative complications in surgical settings. Prior research has demonstrated the benefits of the preoperative SARS-CoV-2 asymptomatic screening test in improving patient outcomes before major surgeries or in areas that are high risk for SARS-CoV-2 [9]. However, the current literature has primarily focused on the clinical benefits. Another report revealed that the implementation of preprocedural SARS-CoV-2 testing might be a low-yield approach with high direct healthcare costs to identify a single patient [10], with limited attention to its cost-effectiveness. Compared with the early days of the pandemic, vaccines have become widely available, and the severity and morbidity of the disease have changed [11]. At the beginning of the pandemic, there was a shortage of personal protective equipment (PPE), and concerns about transmission through high-risk procedures increased; however, as access to PPE improved, these concerns declined. Therefore, the cost-effectiveness of preoperative asymptomatic screening must be reevaluated.

We aimed to investigate the cost-effectiveness of universal preoperative screening in asymptomatic patients for SARS-CoV-2 with polymerase chain reaction (PCR) tests. This investigation helps address the current knowledge gaps and provides evidence-based recommendations for preoperative SARS-CoV-2 testing strategies.

## METHODS

### Model Design

**Figure 1.**
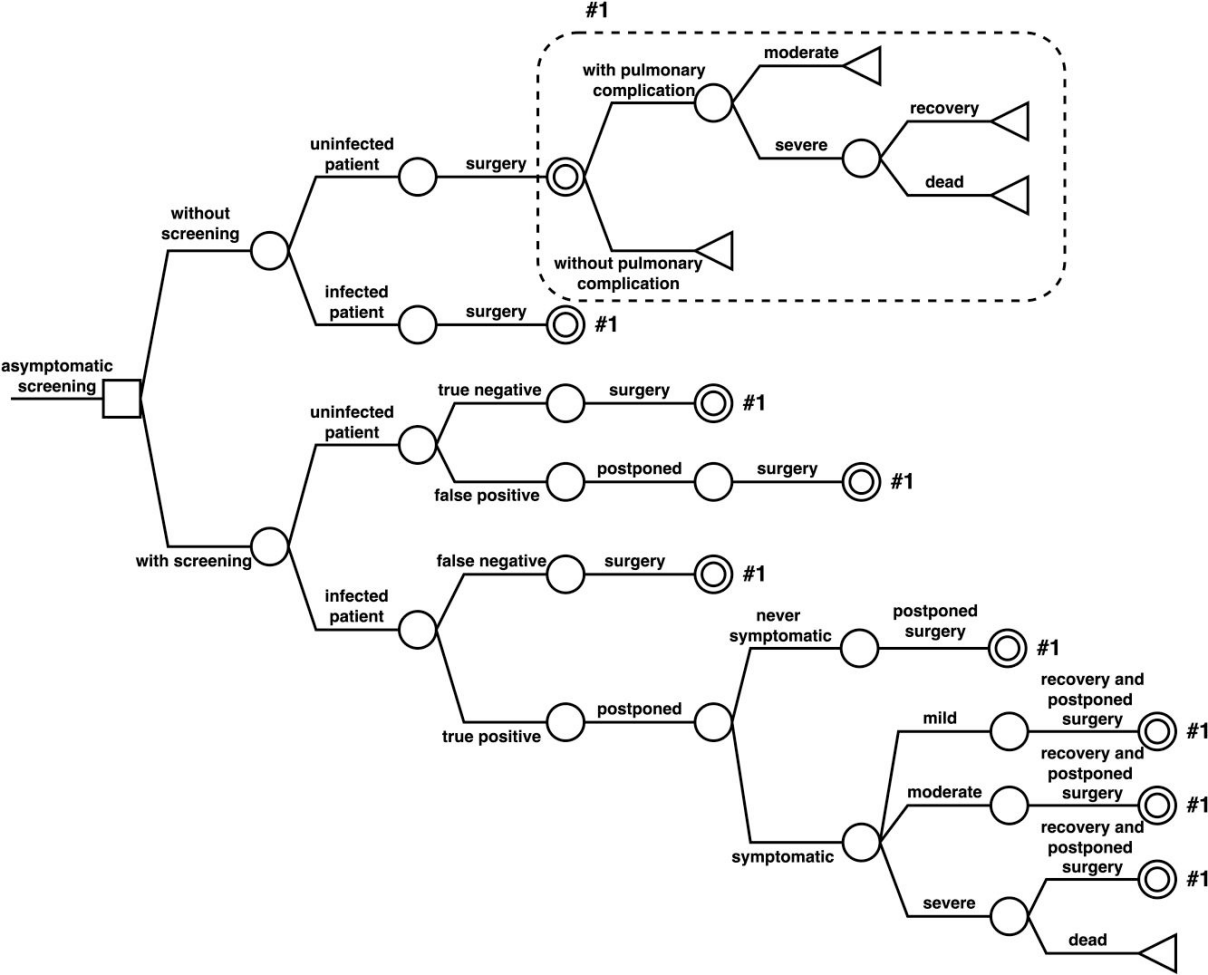
Decision tree used in the analysis. A square decision node indicates a decision point between alternative options. We compared 2 strategies to assess the cost-effectiveness of asymptomatic screening: with asymptomatic screening and without asymptomatic screening. A circle chance node shows points where 2 or more alternative events for a patient are possible. Probabilities are set as the likelihood of a particular event occurring at a chance node. Multiplying probabilities along pathways, moving left to right, estimates the pathways probability. Expected costs and outcomes are calculated as the summation of the pathway values weighted by the pathway probabilities to a triangle terminal node. Clone 1 is the indicated double circle chance node. Severe postoperative pulmonary complications were defined as necessitating invasive mechanical ventilation or acute respiratory distress syndrome.

We developed a decision tree model to evaluate the cost-effectiveness of universal preoperative screening on asymptomatic patients for SARS-CoV-2 with a PCR test (ie, “asymptomatic screening”) from the payer's perspective. The assumed scenario was that a patient without acute symptoms such as fever or upper respiratory tract symptoms would be admitted for scheduled surgery. The evaluation strategy was to perform preoperative screening 2 to 3 days before admission vs no screening (Figure 1). If screening was positive, COVID-19 treatment was implemented, and surgery would be postponed for at least 7 weeks according to a recommendation [12, 13]. The screening test method was PCR with a nasopharyngeal swab sample, the most sensitive of the routinely performed tests. The proportion of PCR-positive asymptomatic patients correlates with the prevalence in the community, and the test-positive rate in asymptomatic patients was assumed to be 0.07% in the base case [14].

This model used a 3-month time horizon; discounting was not performed due to the short time frame. We calculated the incremental cost-effectiveness ratio (ICER) of 1 death averted and per quality-adjusted life-year (QALY) gained. A willingness-to-pay (WTP) threshold was set at 5 000 000 Japanese yen (JPY)/QALY, approximately 38 005 US dollars (USD)/QALY [15]. We used TreeAge Pro 2022 (TreeAge Software, LLC, Williamstown, MA) for the analysis.

### Data Sources

#### Costs

The cost of COVID-19 treatment was based on our previously calculated data, which was based on hospital claims data of patients admitted due to COVID-19 at Keio University Hospital [16]. In the analysis, we defined the surgical cost as the cost of surgical procedures, preoperative cost, and postoperative hospitalization cost without any complications and assumed a flat rate of 1 million JPY (approximately 7601 USD). The screening cost was applied at a price set by the universal fee schedule under the Japanese public healthcare system at the end of 2022, 8500 JPY (64.6 USD). The cost of death from PPCs and COVID-19 was assumed to be equivalent to the cost of survival based on our previous data [16]. We calculated 1 USD as 131.56 JPY at the 2022 mean rate [17].

#### Health Utilities

We extrapolated values widely used in cost-utility analyses of COVID-19 therapeutics as COVID-19 disutility [18, 19], which was initially derived from EuroQOL 5-dimension 3-level index scores collected from patients with *Clostridioides difficile* infection at a French acute-care hospital [20]. We extrapolated the treatment duration for hospitalized COVID-19 to calculate the utility based on our previous data [16].

#### Probabilities and Other Parameters

The model transition probabilities and the sensitivity and specificity of the SARS-CoV-2 PCR test using nasopharyngeal swab specimens were derived from the best available evidence reported in the peer-reviewed literature to date [2, 21–24]. We assumed no reinfection with COVID-19 because the analysis was conducted over a short time horizon of 3 months.

### Modeling Assumptions

Several modeling assumptions were made in the analysis. First, we assumed that the related expenses would be added to the surgical cost if a PPC occurred. Second, due to the lack of appropriate data, the cost of treating PPCs and their treatment duration were assumed to be the same as those of inpatient COVID-19 care, regardless of surgical procedures. Third, utilities were calculated by subtracting the disutility due to a PPC, where the disutility due to COVID-19 was extrapolated from the baseline value; the reduction in utility due to surgery or primary diseases was not considered. Fourth, the baseline value of the utilities in this model was set at 1.0. The baseline health conditions of preoperative patients are very diverse according to their age, the severity of primary diseases, and comorbidities, and the average utility value of preoperative patients was not available. Fifth, we assumed that the utility would return to its baseline level once acute COVID-19 or a PPC had healed. In other words, we did not consider the possibility of suffering from “long COVID” (defined as a multisystemic condition with often severe symptoms following SARS-CoV-2 infection [25]). Sixth, the highly sensitive PCR test can detect residual SARS-CoV-2 for a prolonged period after disease resolution; therefore, a positive test does not equate to active infection or contagiousness. To simplify the model, however, the positive PCR test in the base case was assumed to be the first positive and not the detection of residual SARS-CoV-2 virus.

### Sensitivity Analyses

We performed sensitivity analyses to treat model uncertainty by varying expected parameter values [26]. We performed a deterministic sensitivity analysis and added a 2-way sensitivity analysis on the clinically significant variables between the test-positive rate and the probability of developing a PPC among SARS-CoV-2–positive patients and between test-positive rate and screening cost. The lower limit of the screening cost was set at 4500 JPY (approximately 34.2 USD), the same price as the antigen test for COVID-19; the upper limit was set at 15 000 JPY (approximately 114.02 USD) when the price was first set according to the fee schedule of the Japanese public insurance on 6 March 2020. The lower and upper limits of the test-positive rate in asymptomatic patients were taken from the highest and lowest values in previous reports of screening tests [27, 28]. The lower limit of the probability of developing a PPC in a patient with COVID-19 was set to the probability in a patient without COVID-19, and the upper bound was set to 1 (ie, patients always develop a PPCs). The range of disutility and surgical cost was set at ±50%. The 95% confidence interval (CI) or interquartile range (IQR) was used as the interval estimate for the other parameters, as reported in the literature. When the extant literature did not report the interval estimate, we calculated the 95% CI as necessary using R version 4.1.2 (R Foundation for Statistical Computing, Vienna, Austria).

## RESULTS

Using this decision tree model (Figure 1), we evaluated the cost-effectiveness of the preoperative asymptomatic screening test for COVID-19. Table 1 presents the parameters for the base case analysis with ranges for sensitivity analyses, and Table 2 shows the results of the base case analysis. In the base case analysis, the incremental cost of 1 death averted was 74 469 236 JPY (approximately 566 048 USD), and the ICER per QALY gained was 291 123 368 JPY/QALY (approximately 2 212 856 USD/QALY), above the 5 000 000 JPY/QALY threshold for WTP.

**Table 1.** Input Parameters and Ranges Used for Sensitivity Analyses

Parameter	Base Case Value	Ranges for Sensitivity Analysis		Reference
		Lower Bound	Upper Bound	
Test-positive rate at asymptomatic screening	0.0007	0.0003	0.295	[14, 27, 28]
Sensitivity of SARS-CoV-2 PCR test	0.848	0.768	0.924	[21]
Specificity of SARS-CoV-2 PCR test	0.989	0.974	0.998	[21]
Cost for COVID-19 or PPC treatment, JPY				
Mild	1 113 680	914 748	1 454 288	[16]
Moderate	1 643 909	1 352 123	2 512 986	[16]
Severe	6 210 607	4 755 954	11 234 745	[16]
Cost for SARS-CoV-2 PCR test (screening test), JPY	8500	4500	15 000	
Surgical cost, JPY	1 000 000	500 000	1 500 000	Assumption
Duration of COVID-19 or PPC treatment, d				
Mild	9	7	11	[16]
Moderate	12	10	15	[16]
Severe	17	14	26	[16]
Disutility for COVID-19 or PPC				
Mild	−0.19	−0.285	−0.095	[18]
Moderate	−0.4	−0.6	−0.2	[18, 19]
Severe	−0.6	−0.9	−0.3	[18, 19]
Probability inputs				
Being asymptomatic among SARS-CoV-2 PCR-positive individuals	0.425	0.296	0.778	[22]
Being moderate COVID-19	0.139	0.136	0.142	[24]
Being severe COVID-19	0.047	0.045	0.049	[24]
Mortality with severe COVID-19	0.49	0.469	0.512	[24]
Developing PPC among SARS-CoV-2–positive patients	0.512	0.0416	1	[2]
Developed PPC being severe among SARS-CoV-2–positive patients	0.522	0.48	0.563	[2]
Mortality with severe PPC in SARS-CoV-2–positive patients	0.728^a^	0.674	0.777	[2]
Developing PPC among SARS-CoV-2–negative patients	0.0416	0.0364	0.0473	[23]
Developed PPC being severe among SARS-CoV-2–negative patients	0.308	0.248	0.373	[23]
Mortality with severe PPC in SARS-CoV-2–negative patients	0.333^a^	0.224	0.457	[23]

Abbreviations: COVID-19, coronavirus disease 2019; JPY, Japanese yen; PCR, polymerase chain reaction; PPC, postoperative pulmonary complication; SARS-CoV-2, severe acute respiratory syndrome coronavirus 2.

^a^Because a breakdown of mortality by severity was not available in the literature, we assumed that all mortality was among severely ill patients.

**Table 2.** Results of Base Case Analysis

Outcome	With Screening	Without Screening
Cost, JPY	1 136 083	1 128 254
Incremental cost	7829	(ref)
Mortality	0.00429	0.0044
Decreased mortality	0.000105	(ref)
Utility	0.248306	0.248279
Incremental utility	0.000029	(ref)
Incremental cost of 1 death averted, JPY	74 469 236	…
Incremental cost-effectiveness ratio, JPY/QALY	291 123 368	…

Abbreviations: JPY, Japanese yen; QALY, quality-adjusted life-year.

**Figure 2.**
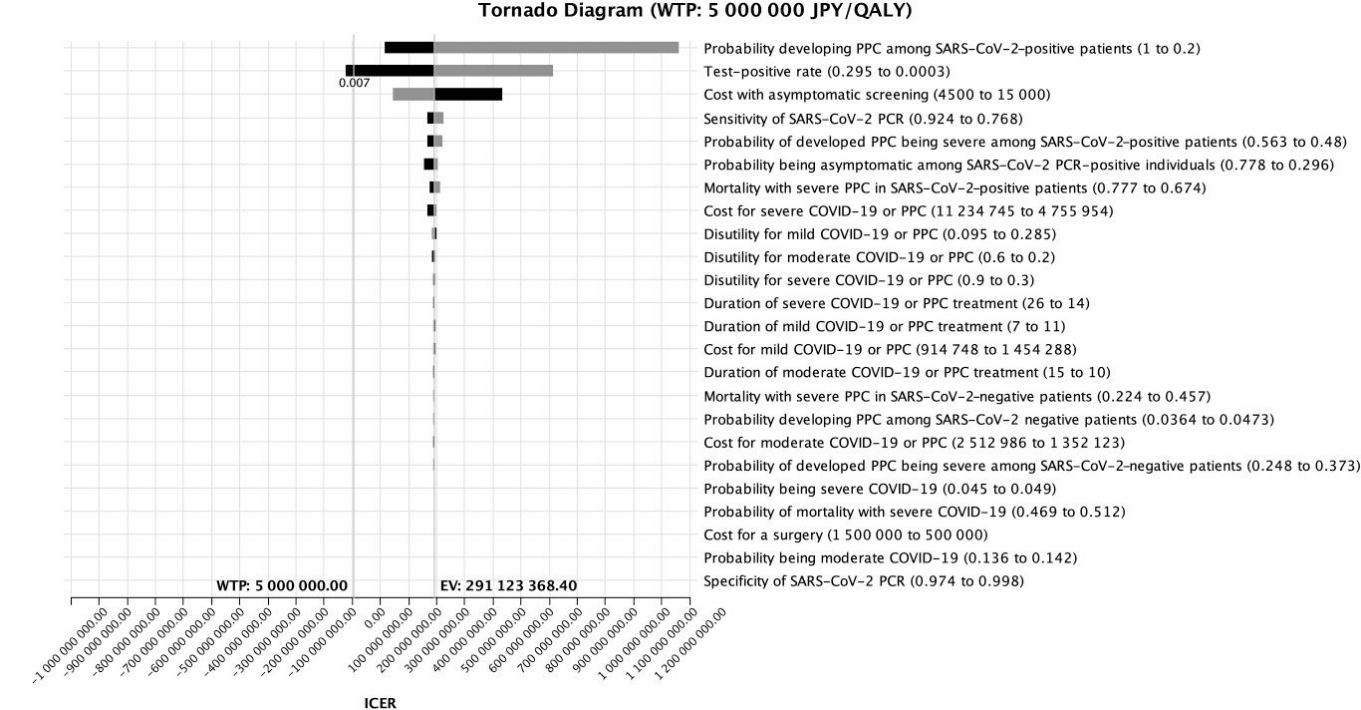
Tornado diagram of sensitivity analysis results. The change in expected value is shown by the black line when the value decreased from the base case analysis and the gray line when the value increased. The ICER fell below the WTP threshold of 5 000 000 JPY/QALY when the test-positive rate exceeded 0.00739 (0.739%). The probability of developing PPCs among SARS-CoV-2–positive patients was treated in a sensitivity analysis with a lower limit of 0.0416. However, as the probability decreased, the ICER diverged to infinity, so this diagram shows the lower limit as 0.2. Abbreviations: COVID-19, coronavirus 2019; EV, expected value; ICER, incremental cost-effectiveness ratio; JPY, Japanese yen; PCR, polymerase chain reaction; PPC, postoperative pulmonary complication; QALY, quality-adjusted life-year; SARS-CoV-2, severe acute respiratory syndrome coronavirus 2; WTP, willingness to pay.

Figure 2 presents the result of sensitivity analyses, showing that the ICER fell below the WTP threshold of 5 000 000 JPY/QALY only when the test-positive rate exceeded 0.00739 (0.739%). A sensitivity analysis was performed on the probability of developing a PPC among SARS-CoV-2–positive patients with a range from 0.0416, equivalent to SARS-CoV-2­negative patients, to 1. However, the ICER diverged to infinity as the probability decreased; thus, the lower limit of this probability is set at 0.2 in Figure 2. The cost of asymptomatic screening was another factor that greatly affected the ICER.

**Figure 3.**
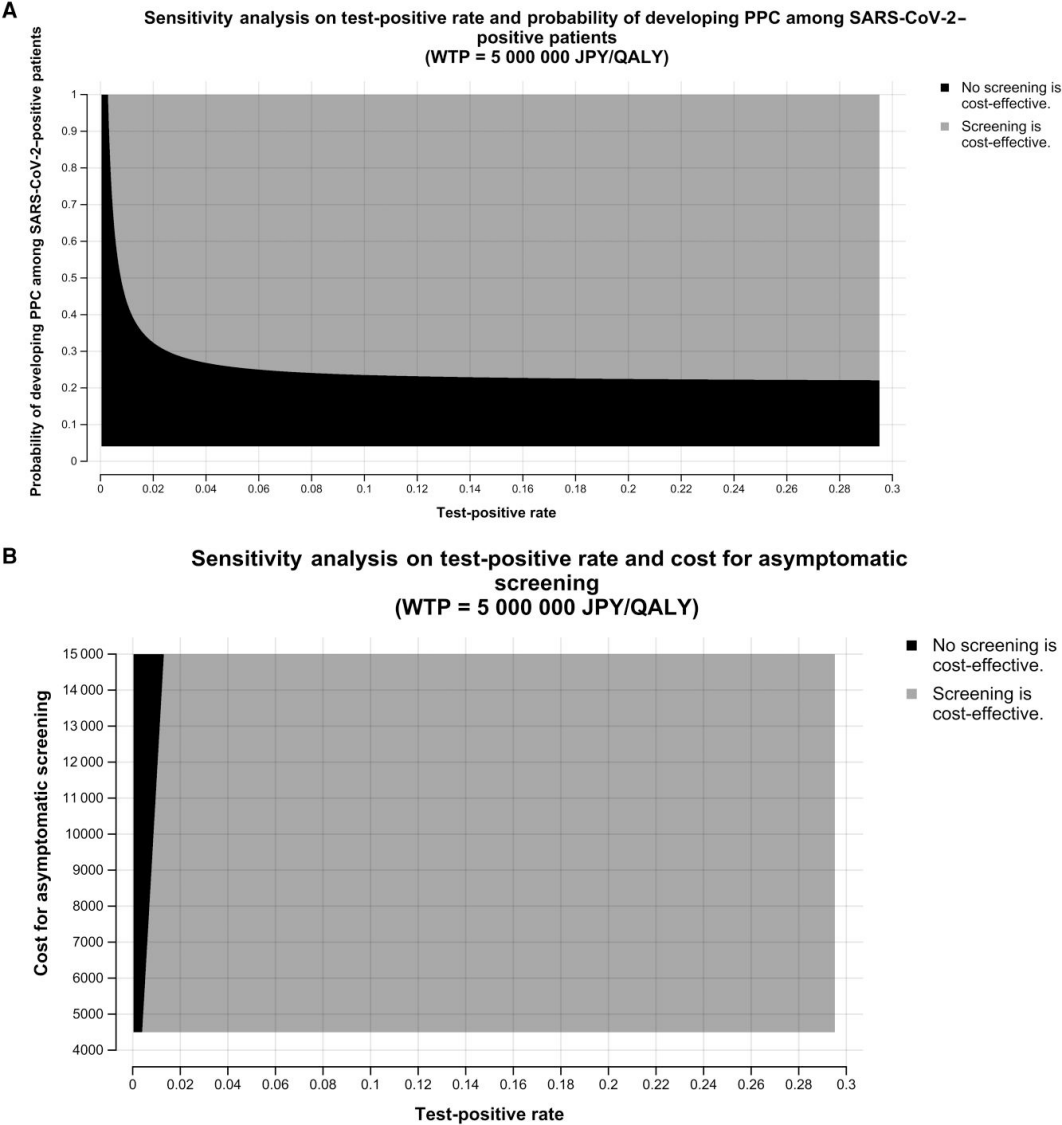
The results of 2-way sensitivity analysis between the test-positive rate and the probability of developing PPCs among SARS-CoV-2–positive patients (*A*) and between the test-positive rate and the screening cost (*B*). The WTP 5 000 000 threshold determines which strategy is more cost-effective under the *X*-axis and *Y*-axis conditions. The black areas represent “without screening is cost-effective”; the gray represents “asymptomatic screening is cost-effective.” Abbreviations: JPY, Japanese yen; PPC, postoperative pulmonary complication; QALY, quality-adjusted life-year; SARS-CoV-2, severe acute respiratory syndrome coronavirus 2; WTP, willingness to pay.

Next, we conducted 2-way sensitivity analyses to investigate the association between the test-positive rate and the probability of developing a PPC among SARS-CoV-2–positive patients (ie, complication rate) and between the test-positive rate and the screening cost (Figures 3*A* , 3*B* ). When the test-positive rate was less than 0.0028 (0.28%), asymptomatic screening was not cost-effective even when the complication rate was 1. As the test-positive rate increased, the cost-effectiveness improved even when the complication rate decreased. However, when the complication rate was 0.3, the test-positive rate needed to be above 0.0256 (2.56%) for the ICER to be below the WTP threshold. When the complication rate was below 0.22, asymptomatic screening was never cost-effective, no matter how high the test-positive rate became.

The test-positive rate needs to be above 0.00391 (0.391%) under a screening cost of 4500 JPY (approximately 34.2 USD), and the test-positive rate needs to be above 0.0130 (1.30%) under a screening cost of 15 000 JPY (approximately 114.02 USD) for the ICER to be below the WTP threshold.

## DISCUSSION

We evaluated the cost-effectiveness of universal preoperative screening of asymptomatic patients for SARS-CoV-2 PCR (ie, “asymptomatic screening”). We showed that the incremental cost of 1 death averted was 74 469 236 JPY (approximately 566 048 USD), and the ICER was 291 123 368 JPY/QALY (approximately 2 212 856 USD/QALY) in the base case analysis, which was far above the 5 000 000 JPY/QALY threshold. ICER fell below the WTP threshold of 5 000 000 JPY/QALY only when the test-positive rate exceeded 0.739% from the sensitivity analysis. The cost-effectiveness varied mainly depending on the test-positive rate, the frequency of PPCs among SARS-CoV-2­–positive patients, and the screening cost. However, no matter how high the test-positive rate, the cost-effectiveness is poor if the probability of developing a PPC among SARS-CoV-2–positive patients is sufficiently reduced.

The expected benefit of asymptomatic screening is to identify those persons who may be infected with SARS-CoV-2 but are unaware of such infection. SARS-CoV-2 is highly contagious and can be transmitted even before the onset of the disease [29], and those asymptomatic infected patients may act as a source of healthcare-associated transmission [30–32]. Therefore, if screening tests before admission could exclude asymptomatic COVID-19 patients, it would be a powerful tool to prevent nosocomial infections. Nonetheless, there is insufficient evidence to determine the usefulness of preadmission asymptomatic screening in preventing nosocomial infections, and the Society for Healthcare Epidemiology of America recommends against routine universal asymptomatic screening to attempt to reduce the risk of nosocomial infections [33]. Honda et al showed that discontinuing asymptomatic screening on admission did not increase nosocomial clusters [34].

Another clinical purpose of asymptomatic screening is to identify an asymptomatic patient before the procedure and to prevent the development of a PPC because of the procedure [9]. Its clinical utility was based on clinical data during the ancestral strain pandemic when neither the vaccine nor the antivirals were available. Robinson et al recently reported that the risk of severe disease and death in patients with the Omicron strain was lower than that in patients with the Delta strain but comparable with that of the ancestral strain [35]. Thus, our results seem valid even in the context of the Omicron strain epidemic. However, widely available vaccines have reduced severity and mortality even during Omicron strain epidemics [11], and oral antivirals are now available for patients with risk factors for progression to severe disease [36, 37]. Robinson et al also reported that vaccines and previous history of infection reduced the risk of severe disease and death [35]. Furthermore, Tande et al revealed that the vaccine also reduced positive rates in preoperative asymptomatic screening [38, 39]. In the current era of widely available vaccines and antivirals, the test-positive rate and the probability of developing PPCs were likely lower than when the ancestral strain was prevalent.

We showed that the cost-effectiveness of asymptomatic screening varied mainly depending on the test-positive rate, the frequency of PPCs, and screening costs. COVID-19 can easily change in severity depending on the emergence of new variant strains and immunity or prevalence. Thus, it is reasonable to determine the implementation of asymptomatic screening according to the severity of the prevalent strain or the community prevalence, which correlates with the test-positive rate. Penney et al proposed the threshold for discontinuation as when the test-positive rate decreased to 1% or less [40]. In our analysis, a test-positive rate of approximately 0.7% or more would suggest that the screening test was cost-effective. However, based on our analysis, we also assume that if the vaccine reduced the incidence of PPCs to the same level as in the pre-COVID-19 era, asymptomatic screening would not be cost-effective, regardless of the test-positive rate.

Our results have some limitations. First, we collected the best available evidence reported in the peer-reviewed literature to date. However, the data concerning the effectiveness of preoperative screening for COVID-19 and the pulmonary complication rate were from when the ancestral strain was prevalent and no vaccine or antiviral treatment was available. When vaccines and antiviral treatments are available under the Omicron strain epidemics, infection with SARS-CoV-2 up to 8 weeks before surgery has been shown not to increase PPCs [41]. Therefore, the cost-effectiveness of asymptomatic screening would be worse than what our analysis showed when vaccines and antiviral treatments were widely available, with our conclusion remaining the same in light of this limitation. Second, this analysis was based on some model assumptions. The cost of treating a PPC and its treatment duration were assumed to be the same as that of inpatient COVID-19 care. However, treatment cost does not significantly affect the results in the sensitivity analysis (Figure 2); therefore, the assumption does not affect the robustness of the results. We set a waiting time of 7 weeks to reschedule surgery if the first PCR test was positive. This was based on data from when the ancestral strain was prevalent; waiting times are likely to have been shorter. However, in this analysis, waiting time does not affect the robustness of the results as it is not associated with changes in costs or outcomes. Further, to simplify the model, the opportunity costs of rescheduling or postponing surgery due to false-positives and potential transmission to others or medical staff due to the absence of screening were not included in the analysis. Third, we analyzed the cost-effectiveness of asymptomatic screening according to Japan's reimbursement system and test-positive rate; therefore, the result must be interpreted in the context of local prevalence and testing costs.

## CONCLUSIONS

Asymptomatic preoperative universal SARS-CoV-2 PCR screening is not cost-effective in the base case analysis. The cost-effectiveness of asymptomatic screening depends mainly on the test-positive rate, the incidence of PPCs, and screening costs. However, if the vaccine reduced the incidence of PPCs to the same level as in the pre–COVID-19 era, asymptomatic screening would not be cost-effective, regardless of the test-positive rate.

## Notes

***Author Contributions.*** S. U.: conceptualization, methodology, formal analysis, investigation, data curation, writing original draft, visualization, and project administration. R. G.: methodology, formal analysis, resources, writing original draft, writing–reviewing and editing, supervision, and project administration. K. H.: methodology, validation, formal analysis, investigation, writing original draft, and writing–reviewing and editing. S. U., Y. U., H. N., A. Y., K. M., and Y. T.: writing–reviewing and editing and project administration. M. M. and Y. K.: writing–reviewing and editing, supervision, and project administration. N. H.: writing–reviewing and editing, supervision, project administration, and funding acquisition.

***Acknowledgments.*** The authors thank Enago (www.enago.jp) for the English language review.

***Ethics approval and consent to participate.*** Personal data were not handled; therefore, based on the Ethical Guidelines for Medical and Health Research Involving Human Subjects issued by the Japanese Ministry of Health, Labour and Welfare, ethics approval did not apply to this study.

***Availability of data and materials.*** The datasets used and analyzed for this study are available from the corresponding author upon reasonable request.
